# Both ghrelin deletion and unacylated ghrelin overexpression preserve muscles in aging mice

**DOI:** 10.18632/aging.103802

**Published:** 2020-07-26

**Authors:** Emanuela Agosti, Marilisa De Feudis, Elia Angelino, Roberta Belli, Maraiza Alves Teixeira, Ivan Zaggia, Edoardo Tamiso, Tommaso Raiteri, Andrea Scircoli, Flavio L. Ronzoni, Maurizio Muscaritoli, Andrea Graziani, Flavia Prodam, Maurilio Sampaolesi, Paola Costelli, Elisabetta Ferraro, Simone Reano, Nicoletta Filigheddu

**Affiliations:** 1 Department of Translational Medicine, University of Piemonte Orientale, Novara, Italy; 2 Department of Health Sciences, University of Piemonte Orientale, Novara, Italy; 3 Division of Oncology, San Raffaele Scientific Institute and Vita-Salute San Raffaele University, Milano, Italy; 4 Molecular Biotechnology Center, Department of Molecular Biotechnology and Health Sciences, University of Torino, Turin, Italy; 5 Department of Translational and Precision Medicine, Sapienza University, Rome, Italy; 6 Department of Public Health, Experimental and Forensic Medicine, Institute of Human Anatomy, University of Pavia, Pavia, Italy; 7 Department of Biomedical Sciences, Humanitas University, Rozzano, Italy; 8 Center for Health Technologies (CHT), University of Pavia, Pavia, Italy; 9 Stem Cell Institute, KU Leuven, Leuven, Belgium; 10 Department of Clinical and Biological Sciences, University of Torino, Turin, Italy; 11 Division of Orthopaedics and Traumatology, Hospital “Maggiore della Carità”, Novara, Italy; 12 Istituto Interuniversitario di Miologia (IIM)

**Keywords:** growth hormone secretagogue receptor, skeletal muscle atrophy, sarcopenia, inflammaging, sarcobesity

## Abstract

Sarcopenia, the decline in muscle mass and functionality during aging, might arise from age-associated endocrine dysfunction. Ghrelin is a hormone circulating in both acylated (AG) and unacylated (UnAG) forms with anti-atrophic activity on skeletal muscle. Here, we show that not only lifelong overexpression of UnAG (Tg) in mice, but also the deletion of ghrelin gene (*Ghrl* KO) attenuated the age-associated muscle atrophy and functionality decline, as well as systemic inflammation. Yet, the aging of Tg and *Ghrl* KO mice occurs with different dynamics: while old Tg mice seem to preserve the characteristics of young animals, *Ghrl* KO mice features deteriorate with aging. However, young *Ghrl* KO mice show more favorable traits compared to WT animals that result, on the whole, in better performances in aged *Ghrl* KO animals. Treatment with pharmacological doses of UnAG improved muscle performance in old mice without modifying the feeding behavior, body weight, and adipose tissue mass. The antiatrophic effect on muscle mass did not correlate with modifications of protein catabolism. However, UnAG treatment induced a strong shift towards oxidative metabolism in muscle. Altogether, these data confirmed and expanded some of the previously reported findings and advocate for the design of UnAG analogs to treat sarcopenia.

## INTRODUCTION

Sarcopenia, the decline in muscle mass and functionality during aging, often results in poor balance, higher risk of falls, fractures, immobilization, loss of independence, and increased morbidity and mortality, thus representing a major health issue in elder individuals [1–3].

Muscle wasting follows the general decline in trophic hormones or in their ability of eliciting their standard cellular response (“resistance”) and the establishment of a chronic mild inflammatory status characteristic of aging [4].

Ghrelin is a gastric hormone peptide circulating in both acylated (AG) and unacylated (UnAG) forms. AG is the endogenous ligand of the growth hormone secretagogue receptor (GHSR-1a), and it is involved in metabolic regulation and energetic balance through induction of appetite, food intake, and adiposity [5, 6]. UnAG does not induce GH release and has no direct effects on food intake, but it shares with AG several biological activities both *in vitro* and *in vivo* independently of the expression of AG receptor. Indeed, *in vivo*, both AG and UnAG protect *Ghsr* KO mice from fasting- and denervation-induced skeletal muscle atrophy, thus proving the existence of a yet unidentified receptor that mediates the common AG and UnAG biological activities [7]. Consistently, both AG and UnAG have direct biological activities *in vitro* on skeletal muscle, including promotion of myoblast differentiation [8], protection from atrophy [7], and stimulation of mitochondrial respiration capacity [9]. In addition, UnAG boosts muscle satellite cell activity, activates autophagy and mitophagy, and stimulates insulin sensitivity, thus promoting muscle regeneration or preventing skeletal mass loss in different disease models, including hindlimb ischemia, high-fat diet-induced diabetes, and chronic kidney disease [10–14].

Similarly to other hormones, in humans, ghrelin levels decrease with aging [15], and the hypoghrelinemic state in the elderly could participate in the establishment of sarcopenia by facilitating the progression of muscle atrophy and limiting skeletal muscle regeneration capability. In mice, on the contrary, it was reported an increase in circulating levels of AG during aging [16], a phenomenon that has been proposed to represent a compensatory mechanism to a decline in receptor or post-receptor functions [17].

The role of ghrelin during aging has been recently investigated through the analysis of ghrelin knockout mice, and, surprisingly, it has emerged that ghrelin deletion prevents age-associated development of obesity and attenuates the decline in muscle strength and endurance seen with aging [18]. Nevertheless, the treatment of old WT mice with AG partially restored muscle performance while increasing food intake and body weight, especially fat mass [18]. Since an increase in obesity, a side effect of AG, in elderly subjects could worsen sarcopenia [19], inducing insulin resistance and diabetes [20], and mediating age-associated adipose tissue inflammation [21], here we explored the potentiality of UnAG in protecting from age-associated muscle wasting avoiding the potential adverse metabolic effects of AG. In addition, we expanded the previously reported characterization of ghrelin knockout mice during aging, especially focusing on the differences between young and aged muscles.

## RESULTS

### Changes of AG and UnAG level changes in WT mice during aging

**Figure 1:**
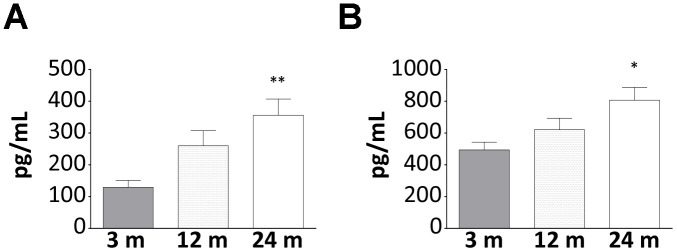
**Increase of AG and UnAG plasmatic levels during aging in WT mice.** Plasmatic levels of AG (**A**) and UnAG (**B**) in 3-, 12-, and 24-month-old mice determined by EIA; 3-month-old mice: N = 5, 12-month-old mice N = 8, 24-month-old mice N = 6. Data in bar graph are presented as mean ± SEM. *p<0.05 and **p<0.01 vs. 3-month-old mice.

Beside the already demonstrated age-related increase of AG [16], in WT mice, we also observed a rise in the circulating levels of UnAG (Figure 1A, 1B), a phenomenon that could represent a compensatory mechanism to age-induced ghrelin resistance [17]. It has been proposed that age-related ghrelin resistance could be due to a decline in ghrelin-elicited signaling or to changes in the levels of its receptor. However, the levels of AG receptor in skeletal muscle were no detectable neither in young nor in old WT animals (data not shown) and, being the receptor mediating the common AG/UnAG antiatrophic activity [7] still unknown, its expression cannot be assessed. Therefore, the relationship between AG/UnAG levels and their receptors still remains to be elucidated.

### Body weight, body composition, and food intake in young, adult, middle aged, and old WT, Tg, and *Ghrl* KO mice

To address whether ghrelin levels during aging affect sarcopenia development, we examined young (3-month-old), adult (6-month-old), middle-aged (12-month-old), and old (24-month-old) Myh6/*Ghrl* transgenic mice characterized by high levels of circulating UnAG, up to 100 times more than WT animals (Tg; [7, 13]), their WT littermates, and ghrelin knockout (*Ghrl* KO; [22]) mice, lacking all the gene-derived peptides.

**Figure 2:**
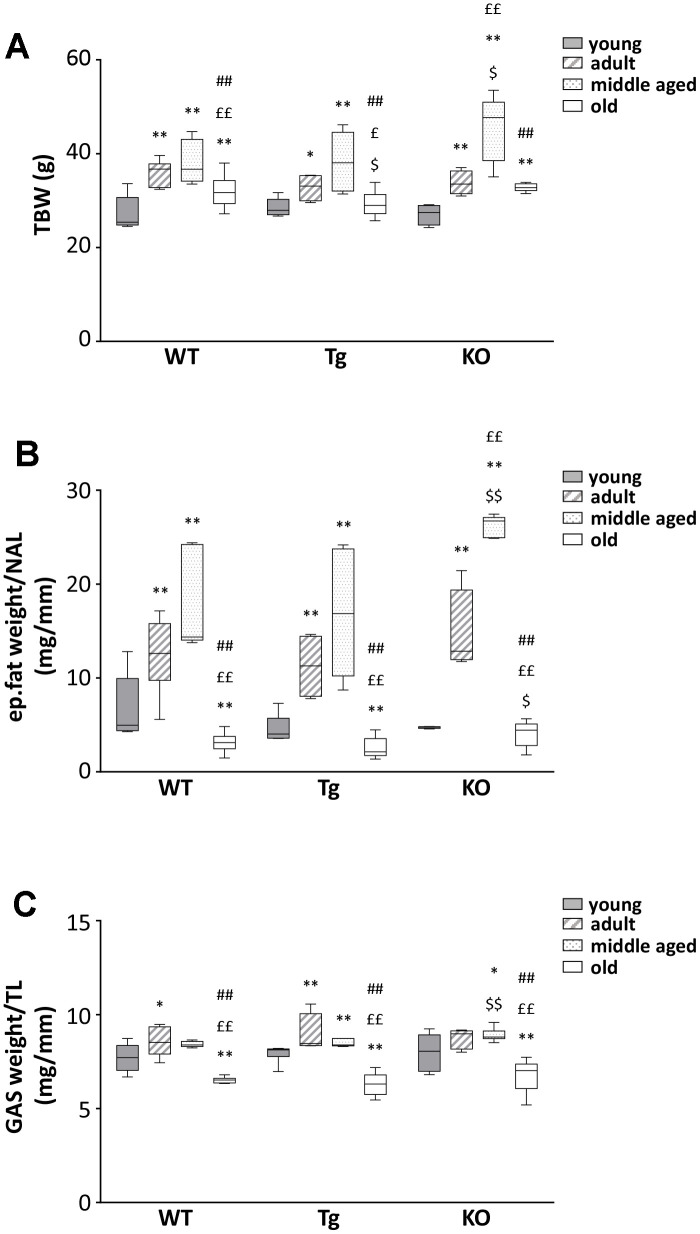
**Body mass composition in young and old WT, Tg, and *Ghrl* KO mice.** (**A**) Total body weight in 3-month old (young), 6-month old (adult), 12-month old (middle aged), and 24-month old (old) mice. (**B**) Epidydimal fat mass normalized to nose-to-anus length (NAL) and (**C**) gastrocnemius weight normalized to the tibial length (TL). Young mice: WT = 6, Tg = 7, *Ghrl* KO = 5; adult mice: WT = 7, Tg = 4, *Ghrl* KO = 5; middle aged: WT = 5, Tg = 6, *Ghrl* KO = 9; old mice: WT= 13, Tg= 14, *Ghrl* KO = 11. Data in bar graphs are presented as mean ± SEM. For each box plot, the lower and up boundaries denote the 25^th^ and the 75^th^ percentile of each data set, respectively, the horizontal line represents the median, and the whiskers represent the min and max of values. *p<0.05 and **p<0.01 vs. young mice; ^£^p<0.05 and ^££^p<0.01 vs. adult mice; ^#^p<0.05 and ^##^p<0.01 vs. middle aged mice; ^$^p<0.05 and ^$$^p<0.01 Tg and *Ghrl* KO vs. WT.

We observed a general increase in body weight and BMI for all genotypes during aging up to 12 months, with a remarkable drop at 24 months of age (Figure 2A and Table 1). A similar trend was observed also for epididymal fat and skeletal muscles (Figure 2B, 2C and Table 1), suggesting the establishment of progressive sarcopenia starting from 12 months in all genotypes.

**Table 1:** Phenotypical characterization of WT, Tg, and *Ghrl* KO mice at different ages.

		**3 months (young)**	**6 months (adult)**	**12 months (middle aged)**	**24 months (old)**
BMI (g/cm^2^)	WT	3.34±0.18	3.88±0.08^*^	3.97±0.07^**^	3.88±0.11^**^
	Tg	3.30±0.12	3.81±0.14^*^	4.16±0.21^**^	3.71±0.07^**,#^
	KO	3.77±0.16	3.61±0.12	4.75±0.14^**,$$,££^	3.68±0.09^##^
nose-to-anus length (NAL, mm)	WT	90.25±0.35	95.26±1.26^**^	95.68±1.42^**^	90.66±1.23^££,#^
	Tg	91.07±1.49	93.87±1.75	95.76±1.01^*^	89.56±1.33^##^
	KO	85.11±0.76^$$^	96.52±1.74^**^	97.96±1.57^**^	93.06±0.89^**,#^
tibial length (TL, mm)	WT	19.84 ± 0.09	20.47 ± 0.24	20.48 ± 0.13^**^	20.49 ± 0.17^**^
	Tg	19.85 ± 0.11	20.50 ± 0.18^**^	20.70 ± 0.11^**,$$^	20.07 ± 0.10^**,$$,£^
	KO	19.82 ± 0.09^$^	20.10 ± 0.07^*^	20.27 ± 0.18	20.70 ± 0.32^**,££^
*tibialis anterior*/TL (mg/mm)	WT	2.38±0.02	2.54±0.07	2.56±0.01^**^	2.28±0.03^££,##^
	Tg	2.52±0.04^$^	2.5±0.02	2.49±0.09	2.23±0.04^**,££,#^
	KO	2.65±0.14	2.95±0.08^*,$$^	2.81±0.04^$$^	2.38±0.06^££,##^
quadriceps/TL (mg/mm)	WT	10.1±0.36	11.74±0.38^**^	10.79±0.16^£^	7.98±0.25^**,££,##^
	Tg	10.20±0.45	10.87±0.2	10.34±0.42	7.97±0.12^**,££,##^
	KO	11.28±0.57	12.36±0.42	11.64±0.08^$$^	8.50±0.36^**,££,##^
*extensor digitorum longus*/TL (mg/mm)	WT	0.56±0.02	0.58±0.02	0.56±0.02	0.48±0.01^**,££,##^
	Tg	0.51±0.03	0.51±0.01^$$^	0.58±0.01^**,££^	0.48±0.01^##^
	KO	0.55±0.03	0.61±0.03	0.61±0.01^*^	0.47±0.02^*,££,##^
soleus/TL (mg/mm)	WT	0.45±0.02	0.46±0.03	0.48±0.01	0.38±0.01^**,££,##^
	Tg	0.46±0.01^$$^	0.47±0.03	0.46±0.02	0.36±0.01^**,££,##^
	KO	0.43±0.02^$$^	0.49±0.01^**^	0.56±0.02^**,££^	0.36±0.02^**,$$,££,##^
heart weight/NTA (mg/mm)	WT	1.34±0.04	1.62±0.07^**^	1.56±0.09^**^	2.00±0.10^**,££,#^
	Tg	1.40±0.08	1.35±0.03^$$^	1.47±0.04	2.14±0.11^**,££,##^
	KO	1.38±0.14	1.37±0.02^$$^	1.40±0.04^$$^	1.92±0.11^**,££,##^
liver weight/NTA (mg/mm)	WT	14.44±0.63	15.75±1.14	17.31±0.71^**^	19.44±0.52^**,££,#^
	Tg	15.64±0.48	15.14±0.77	18.21±1.35	20.55±0.64^**,££,#^
	KO	14.64±0.81	15.55±0.66	18.55±1.2^*^	18.13±0.94^**^
spleen weight/NTA (mg/mm)	WT	0.81±0.04	1.07±0.10^*^	0.91±0.05^*,££^	1.91±0.27^*,£^
	Tg	0.84±0.04^$$^	0.89±0.05	0.84±0.02	1.67±0.11^**,££,##^
	KO	0.83±0.05^$$^	0.84±0.04	1.04±0.06^*,$$,££^	1.29±0.14^*,£^
daily food intake (g)	WT	4.11±0.11	4.56±0.11^**^	4.23±0.10^£^	4.18±0.23
	Tg	3.96±0.11	4.78±0.04^*^	4.21±0.25^£^	4.39±0.21^££^
	KO	4.33±0.23^$^	4.26±0.19	4.33±0.17	4.21±0.14

Young (WT = 6, Tg = 7, *Ghrl* KO = 5), adult (WT = 7, Tg = 4, *Ghrl* KO = 5), middle aged (WT = 5, Tg = 6, *Ghrl* KO = 9), old (WT = 13, Tg = 14, *Ghrl* KO = 11) mice were sacrificed and measurements of the indicated parameters were carried out as described in Materials and Methods. Muscle mass and tibial length values were calculated as the mean of right and left hindlimbs. Values are the means ± SEM; *p<0.05 and **p<0.01 vs. young mice; ^£^p<0.05 and ^££^p<0.01 vs. adult mice; ^#^p<0.05 and ^##^p<0.01 vs. middle aged mice; ^$^p<0.05 and ^$$^p<0.01 Tg and *Ghrl* KO vs. WT.

Other organs, such as the heart, liver, and spleen, on the contrary, steadily increased during aging (Table 1). Daily food intake increased in adult mice compared to young ones in all genotypes. Afterwards, in WT and Tg, daily food intake decreased with aging. On the contrary, *Ghrl* KO mice exhibited a higher food intake at 3 months of age that remained stable during aging (Table 1).

### Muscle and cognitive performance in young and old WT, Tg, and *Ghrl* KO mice

**Figure 3:**
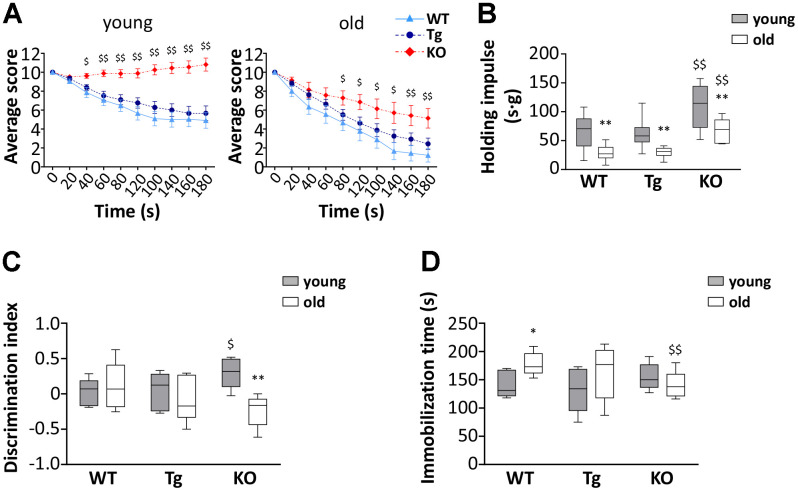
**Muscle functionality and behavior in young and old WT, Tg, and *Ghrl* KO mice.** (**A**) Average score trend in hanging wire test of 3-month old (young) and 24-month old (old) WT, Tg, and *Ghrl* KO mice and (**B**) average holding impulse in the same test. Young mice: WT = 21, Tg = 21, *Ghrl* KO = 27; old mice: WT = 9, Tg = 16, *Ghrl* KO = 7. *p<0.05 and **p<0.01 vs WT. (**C**) Memory was evaluated through the object recognition test and expressed as a discrimination index between a familiar and a novel object (seconds on novel – seconds on familiar)/(seconds on novel + seconds on familiar). (**D**) Anxiety was assessed as the time spent immobile during 6 minutes of tail suspension. Young mice: WT = 5, Tg = 5, *Ghrl* KO = 6; old mice: WT = 6, Tg = 7, *Ghrl* KO = 6. For each box plot, the lower and up boundaries denote the 25^th^ and the 75^th^ percentile of each data set, respectively, the horizontal line represents the median, and the whiskers represent the min and max of values. *p<0.05 and **p<0.01 old vs. young; ^$^p<0.05 and ^$$^p<0.01 Tg and *Ghrl* KO vs. WT.

To assess the effect of altered ghrelin levels during aging on muscle functionality, we measured the performances of young and old WT, Tg, and *Ghrl* KO mice using the hanging wire test. This test allows determining muscle function and coordination by counting the times a mouse falls or reaches one of the sides of the wire and the tension that the animal develops for maintaining itself on the wire, against gravity, by measuring the longest suspension time. In general, mice from all genotypes showed a decrease in muscle performance during aging. However, surprisingly, *Ghrl* KO mice outclassed both WT and Tg mice, at both ages, in the “falls and reach” score, as well in the holding impulse (Figure 3A, 3B). On the other hand, Tg mice performances were barely distinguishable from those of their WT littermates, both in young and old animals.

Since the score on the hanging wire test could be affected by cognitive performance and stress susceptibility, we assessed if cognitive capability and anxiety/depression of mice were different among the strains. Young *Ghrl* KO mice undergoing the object recognition test obtained a higher discrimination index than young WT and Tg (Figure 3C). However, at 24 months of age, while no significant changes were observed in the discrimination index of WT and Tg mice, that of *Ghrl* KO mice dropped to a level similar to those of WT mice. The tail suspension test, with the time spent immobile representing a measure of anguish, showed no differences in the immobility time of young mice (Figure 3D). During aging, both WT and Tg mice had a propensity to develop depression-like behaviors, while *Ghrl* KO mice showed substantial preservation of the immobility time during aging.

### Skeletal muscle atrophy during aging in WT, Tg, and *Ghrl* KO mice

**Figure 4:**
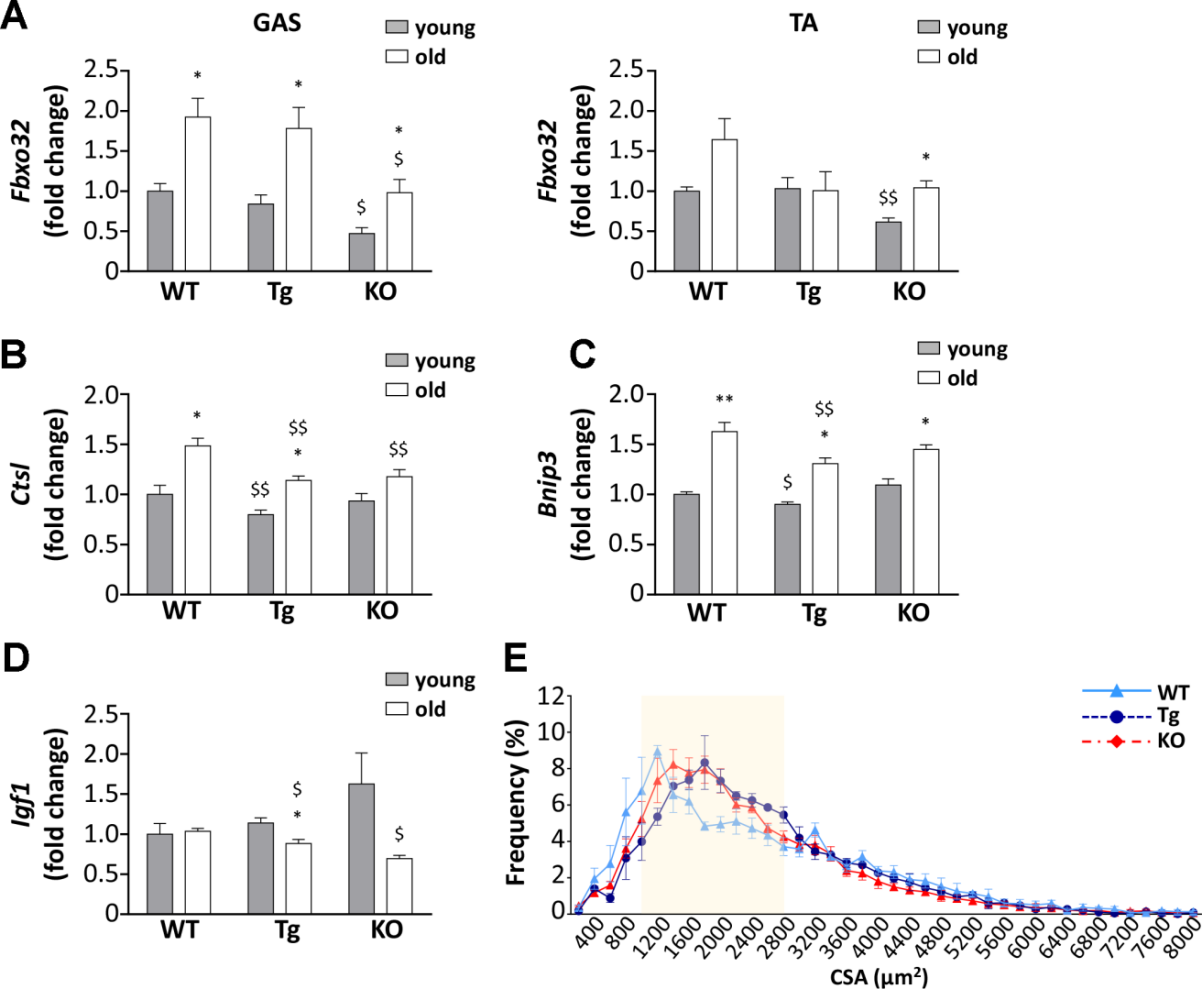
**Ghrelin peptides effects on sarcopenia-dependent atrophy.** (**A**) Atrogin-1 (*Fbxo32*) expression in gastrocnemius (GAS) and *tibialis anterior* (TA) of 3-month old (young) and 24-month old (old) mice determined by real-time RT-PCR. In GAS, young mice: WT = 4, Tg = 4, Ghrl KO = 4; old mice: WT = 4, Tg = 3, Ghrl KO = 4. In TA, young mice: WT = 4, Tg = 3, Ghrl KO = 4; old mice: WT = 8, Tg = 6, Ghrl KO = 5. (**B**) Cathepsin-L (*Ctsl*), (**C**) Bnip-3 (*Bnip3*), and (**D**) IGF-1 (*Igf1*) expression in gastrocnemius of 3-month old (young) and 24-month old (old) mice determined by real-time RT-PCR. Young mice: WT = 3, Tg = 3, Ghrl KO = 4; old mice: WT = 7, Tg = 9, Ghrl KO = 5. (**E**) Cross-sectional area (CSA) frequency distribution of myofibers in GAS of old WT, Tg, and *Ghrl* KO mice. WT= 4, Tg= 3, *Ghrl* KO= 5. The shadowed area of the graph represents the section of statistically significant differences among curves. Data are presented as mean ± SEM. *p<0.05 and **p<0.01 old vs. young; ^$^p<0.05 and ^$$^p<0.01 Tg and *Ghrl* KO vs. WT.

Despite the lack of significant differences in the weight of muscles among the three genotypes and the general decrease in muscle mass in aged mice (Figure 2C and Table 1), a closer examination of muscles revealed differences in the expression of Atrogin-1 (Fbxo32), a marker and a mediator of muscle atrophy, during aging [23]. The age-dependent increase of Atrogin-1 mRNA expressions was remarkable in gastrocnemii of WT and Tg mice and was milder in *Ghrl* KO mice (Figure 4A), although it should be considered that Atrogin-1 mRNA levels were basally lower in young *Ghrl* KO mice compared to the other genotypes. This was also true in *tibialis anterior* (TA) muscles, where Atrogin-1 was significantly increased during aging only in *Ghrl* KO mice, reaching the values of young WT and Tg animals. Conversely, the increase of Atrogin-1 during aging was not significant in WT and absent in Tg animals. The mRNA expression of other markers of autophagic-mediated catabolism, such as cathepsin-L and Bnip-3, showed a general tendency to increase with aging in all genotypes (Figure 4B, 4C), although the increase in cathepsin-L was significant only in WT and Tg mice. In the aged groups, cathepsin-L gene expression in both Tg and *Ghrl* KO mice was reduced compared to the WT animals, while Bnip-3 levels significantly decreased only in Tg mice. On the contrary, the expression of the anabolic marker IGF1 showed the general tendency to decrease with aging in Tg and *Ghrl* KO mice, although the reduction was significant only in the former (Figure 4D). Nevertheless, in old animals, *Ghrl* KO mice displayed a statistically significant lower expression of IGF1 compared to WT mice. Analyzing the fiber cross-sectional area distributions of gastrocnemius (GAS) muscles from young adult and old WT mice, it was evident a clear shift toward smaller areas in the latter (Supplementary Figure 1). Since the muscles of young Tg and KO mice, in unstimulated conditions, do not differ from those of WT animals [13, 22], the shift towards larger areas in old Tg and *Ghrl* KO muscles compared to old WT muscles (Figure 4E) indicates that both high levels of circulating UnAG and the lack of ghrelin peptides protect against muscle mass loss during aging.

### Muscle fiber types and metabolism in old WT, Tg, and *Ghrl* KO mice

**Figure 5:**
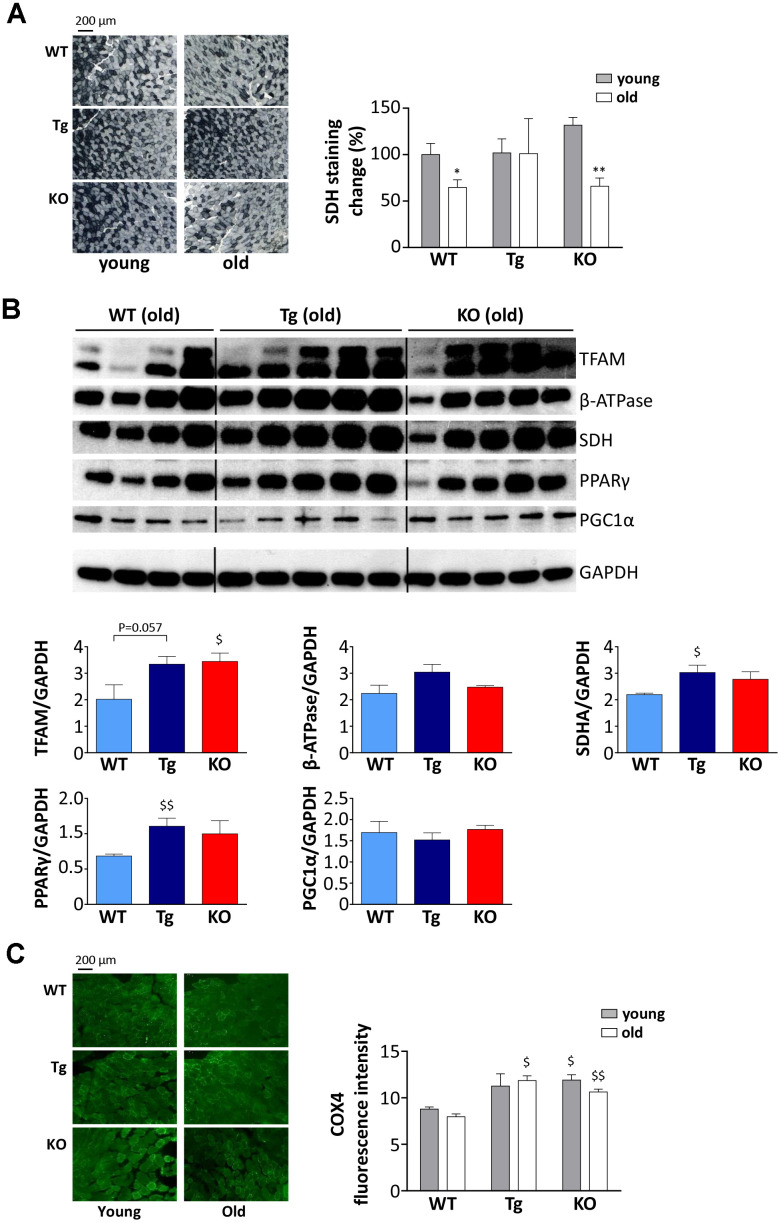
**Metabolic shift in TA muscles of WT, Tg, and *Ghrl KO* mice.** (**A**) Representative images of succinate dehydrogenase (SDH) staining (scale bars: 200 μm) representing the oxidative capacity of TA muscles of 3-month old (young) and 24-month old (old) mice and quantification of SDH-positive fibers in TA muscle presented as the percentage of SDH-positive area above the total muscle surface. Young mice: WT= 5, Tg= 4, *Ghrl* KO= 5; old mice: WT= 4, Tg= 3, *Ghrl* KO= 5. (**B**) Representative Western blots images and protein densitometry quantification for mitochondrial transcription factor A (TFAM), ATP-synthase β-subunit (β-ATPase), succinate-dehydrogenase complex subunit-A (SDHA), peroxisome proliferator-activated receptor gamma (PPARγ), and PPARγ-coactivator-1-α (PGC1α) protein levels. Protein levels were probed in gastrocnemii of old mice, WT= 4, Tg= 5, *Ghrl* KO= 5. (**C**) Representative images of cytochrome c oxidase subunit 4 (COX4) immunofluorescence (scale bars: 200 μm) and quantification. Young mice: WT = 3, Tg = 3, Ghrl KO = 3; old mice: WT = 4, Tg = 4, Ghrl KO = 4. Data are presented as mean ± SEM. *p<0.05 and **p<0.01, old vs. young; ^$^p<0.05 and ^$$^p<0.01, Tg and *Ghrl* KO vs. WT.

Muscles are more susceptible/resistant to atrophy also depending on their fiber type composition, being glycolytic fibers more prone to undergo mass loss compared to the oxidative ones [24]; therefore, we assessed if high levels of UnAG or the lack of ghrelin peptides could affect muscle metabolism. Succinate-dehydrogenase (SDH) staining revealed that oxidative ability, *i.e.*, the activity of complex II of the electron transport chain, decreases with aging both in WT and *Ghrl* KO mice, whereas it was maintained in Tg (Figure 5A), suggesting that UnAG stimulates an oxidative metabolism, counteracting the age-associated shift from oxidative to glycolytic metabolism.

Since aging muscles display mitochondrial dysfunction [25], we assessed the expression levels of mitochondria and oxidative metabolism markers such as mitochondrial transcription factor A (TFAM), ATP-synthase β-subunit (β-ATPase), SDH subunit-A (SDHA), peroxisome proliferator-activated receptor gamma (PPARγ), and PPARγ-coactivator-1-alpha (PGC1α). In line with the results reported in Figure 5A, we observed an up-regulation of SDHA in old Tg mice compared to WT (Figure 5B). In addition, old Tg mice showed an increase in PPARγ. On the other hand, β-ATPase and PGC1α, a master regulator of mitochondrial biogenesis, did not show different expression levels among the three genotypes. Yet, *Ghrl* KO mice showed an up-regulation of TFAM, another marker of mitochondrial biogenesis. Coherently, the mitochondrial content in muscles, assessed as the quantity of cytochrome c oxidase subunit 4 (COX4) in immunofluorescence, was higher in *Ghrl* KO mice than in WT, both in young and old animals (Figure 5C). Of note, also old Tg animals had significantly higher levels of COX4 compared to WT.

### Inflammation markers in old WT, Tg, and *Ghrl* KO mice

**Figure 6:**
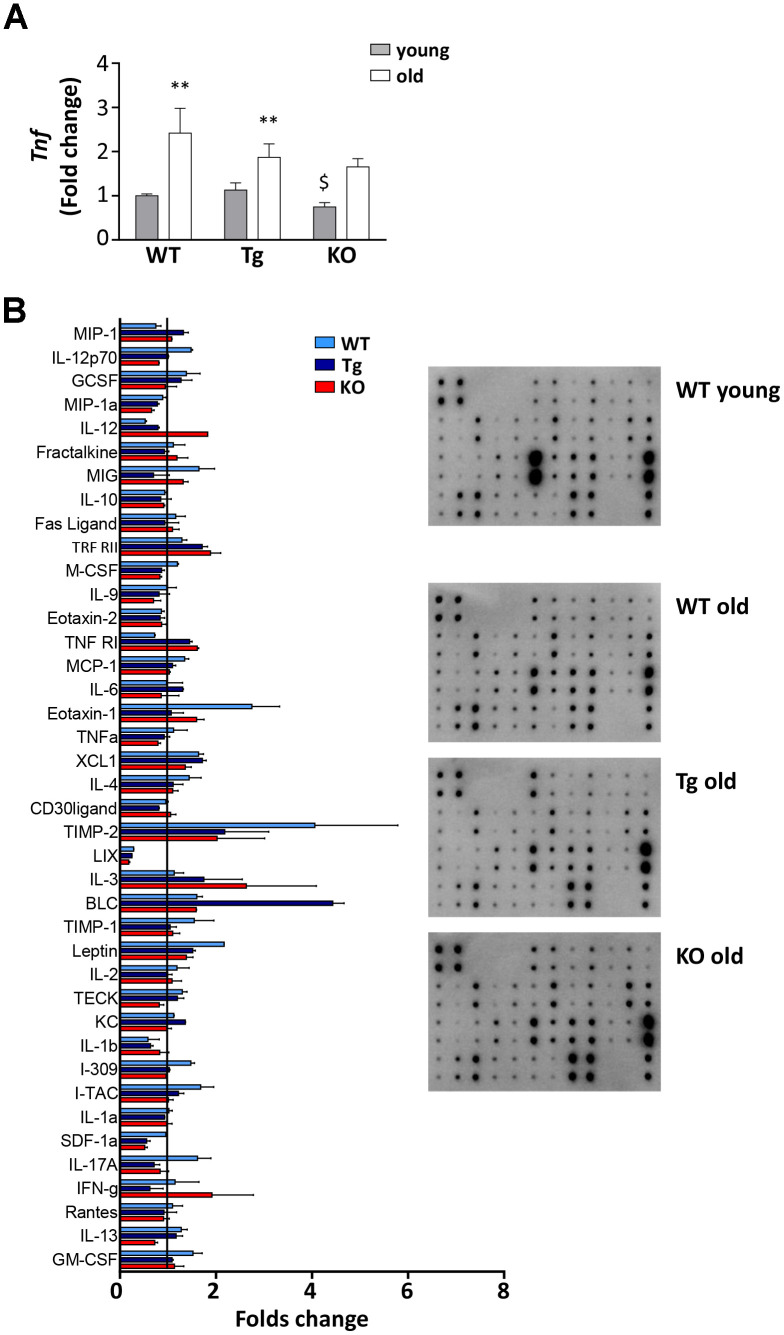
**Inflammatory response in old WT, Tg, and *Ghrl KO* mice.** (**A**) TNF-α (*Tnf*) expression in epididymal fat of 3-month old (young) and 24-month old (old) WT, Tg, and *Ghrl* KO mice, determined by real-time RT-PCR. Data in bar graph are presented as mean ± SEM. Young mice: WT= 3, Tg= 4, *Ghrl* KO = 4; old mice: WT= 5, Tg= 7, *Ghrl* KO = 5. (**B**) Serum biomarkers involved in systemic inflammation normalized on the levels in young WT mice (reference line). Young mice WT=4; old mice: WT= 4, Tg= 4, *Ghrl* KO = 4 pulled into two groups. Data in bar graph are presented as mean ± SD. *p<0.05 and **p<0.01, old vs. young; ^$^p<0.05 Tg and *Ghrl* KO vs. WT.

With age, the spleen undergoes enlargement and disorganization of its architecture that can lead to age-related decline of immune function and chronic inflammation, that, in turn, could contribute to the decrease in muscle mass and functionality [26–28]. All three genotypes showed a significant increase of spleen weight with age, although to a lesser extent in *Ghrl* KO mice (Table 1). Adipose tissue as well contributes to the establishment of age-associated systemic inflammation, as it becomes dysfunctional with age, increasing the production of pro-inflammatory cytokines while decreasing the anti-inflammatory ones [29]. TNFα mRNA levels in epididymal fat increased during aging in all the genotypes, but to a lesser extent in Tg and *Ghrl* KO mice (Figure 6A), confirming the low-grade systemic inflammation in these mice. The impact of ghrelin peptide levels on age-related systemic inflammation was explored through an antibody-spotted array directed against several circulating markers of inflammation on pooled samples (Figure 6B and Supplementary Figure 2). As expected, old WT mice showed a general tendency to increase pro-inflammatory markers and to decrease the anti-inflammatory ones compared to young mice (reference line in Figure 6B). By contrast, a trend towards reduction could be observed in both old Tg and *Ghrl* KO mice compared to old WT for some pro-inflammatory markers, such as SDF1α, eotaxin-1 (CCL11), I-309 (CCL1), leptin, M-CSF, and IL-12p70, seemed to be lower. Conversely, the anti-inflammatory soluble TNF-R1 gave the impression of being increased in both Tg and *Ghrl* KO mice.

### UnAG pharmacological treatment improved the physical performance of old WT mice by inducing a metabolic switch in muscles

**Figure 7:**
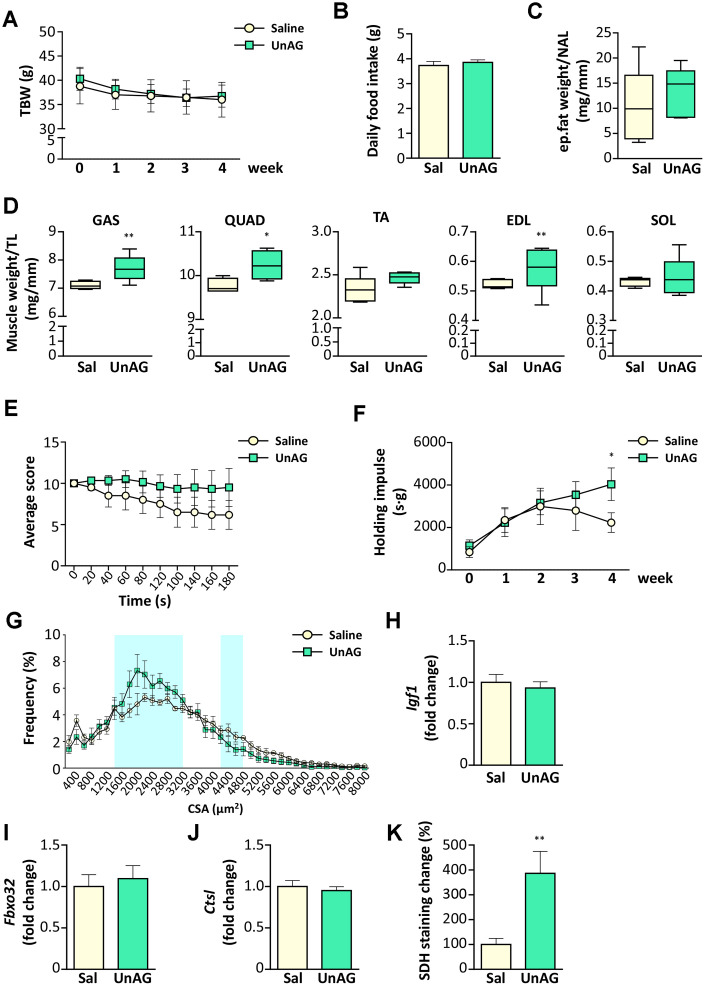
**Exogenous administration of UnAG in old WT mice attenuates age-related muscle decline.** UnAG 100 μg/Kg was injected daily i.p. for 28 days to 18-month-old mice (N = 6). Age- and weight-matched controls were injected with saline (N =6). (**A**) Total body weight; (**B**) daily food intake; (**C**) change in epidydimal fat mass normalized to nose-to-anus length (NAL) and in (**D**) muscle mass normalized to the tibial length (TL). GAS: gastrocnemius, SOL: soleus, QUAD: quadriceps, TA: tibialis anterior, EDL: extensor digitorum longus. (**E**) Average score trend in hanging wire test and (**F**) average holding impulse. (**G**) Cross-sectional area (CSA) frequency distribution of myofibers in GAS. The shadowed areas of the graph represent the sections of statistically significant differences among curves. (**H**) IGF-1 (*Igf1*), (**I**) Atrogin-1 (*Fbxo32*), and (**J**) Cathepsin-L (*Ctsl*) expression in gastrocnemius determined by real-time RT-PCR. (**K**) Quantification of SDH staining in TA muscle presented as the percentage of SDH-positive area above the total muscle surface. Data in bar graph are presented as mean ± SEM. For box plots, the lower and up boundaries denote the 25^th^ and the 75^th^ percentile of each data set, respectively, the horizontal line represents the median, and the whiskers represent the min and max of values. *p<0.05 and **p<0.01 vs. saline-treated controls.

Old WT mice were treated with UnAG (100 μg/kg intraperitoneally once a day) for 28 days and compared to age- and weight-matched controls treated with saline. UnAG administration had no significant effect on total body weight (Figure 7A), daily food intake (Figure 7B), and epidydimal fat (Figure 7C). On the other hand, muscle weights showed a tendency towards higher values in UnAG-treated mice that were significant in gastrocnemii, quadriceps, an d EDL muscles (Figure 7D). UnAG progressively increased the physical performance of mice, seen as “falls and reach” score and holding impulse in the hanging wire test (Figure 7E, 7F). The difference in the holding impulse in UnAG-treated mice reached statistical significance at 28 days. However, at the end of the experimental period, there was no shift towards larger cross-sectional areas of gastrocnemius fibers, although the shape of UnAG-treated muscle distribution showed an increment of middle-sized fibers (Figure 7G). Despite the increase in muscle weight (Figure 7D), UnAG did not alter the expression of IGF-1 (Figure 7H). Furthermore, UnAG treatment was unable to decrease the expression of Atrogin-1 and cathepsin-L (Figure 7I, 7J). Since the better muscle performance could derive from a shift in muscle metabolism, we assessed SDH activity in gastrocnemii and observed that, indeed, UnAG induced a substantial increase in SDH staining in muscles (Figure 7K).

## DISCUSSION

The decline in muscle mass, functionality, and physical performance distinctive of aging can be ascribed, at least in part, to age-associated endocrine and metabolic dysfunction. Among the dysregulated hormones, in humans, it has been observed reduced circulating levels of ghrelin and decreased expression on the cell surface of its receptor GHSR-1a [17] that may contribute to sarcopenia by facilitating the progression of muscle atrophy and limiting skeletal muscle regeneration capability. No changes were reported for UnAG in elderly compared to young people [30], although the broad age range (21-65 years) of the “young” group could have leveled any difference. In mice, on the contrary, it was reported an increase in circulating levels of AG during aging and no changes in UnAG [16]. However, together with the age-related increase of AG, we also observed a rise in the circulating levels of UnAG (Figure 1A, 1B), possibly owing to the different methods used compared to the previous work. The increase of UnAG could represent a compensatory mechanism to a decline in receptor or post-receptor functions, as previously suggested for AG [17]. However, the undetectable levels of *Ghsr* in skeletal muscles and the absence of a known receptor for UnAG prevented us from demonstrating such a hypothesis in this specific context. Still, the age-related increase in AG and UnAG levels may be part of an adaptive response to a negative energy balance and suggests that these peptides could be involved in the aging process for the many activities that characterize each or both forms of ghrelin peptide, including the protection from muscle wasting (AG and UnAG; [7]). Muscle atrophy is indeed one of the main degenerative processes involved in sarcopenia and, for the above-mentioned anti-atrophic activity of AG and UnAG, it was reasonable to expect that high circulating levels of UnAG, as in the Tg mice used in this study, would result in less sarcopenia by protecting from muscle wasting. This was actually the case, being Tg muscles partially resistant to atrophy during aging (Figure 4A, 4E).

On the contrary, the finding that old *Ghrl* KO mice were atrophy-resistant, featuring enhanced physical performance (Figure 3A, 3B), lower levels of catabolism-related genes (Figure 4A–4C), and larger myofiber areas than WT mice (Figure 4E), was less expected, as we have formerly demonstrated that, upon a traumatic event, muscle regeneration in *Ghrl* KO mice is reduced, due to an impaired satellite cell self-renewal activity [22]. Also, old *Ghrl* KO mice are more susceptible to fasting-induced muscle atrophy likely because of an altered mitochondrial function [9]. Nevertheless, most of our findings on aged *Ghrl* KO mice are in accordance with the previous demonstration that ghrelin gene deletion results in the prevention of the age-associated decline in muscle strength and endurance [18]. Yet, we also obtained some different results compared with the previously published ones. For a start, we observed an increase in body weight with aging in both WT and *Ghrl* KO mice (Figure 2A and Table 1), without differences in old *Ghrl* KO compared to old WT. However, this divergence could arise from the different caging conditions used, as individually-housed animals eat more and move less than animal housed in groups, thus leading, in the long run, to significant body weight differences. More strikingly, although 24-month-old mice are significantly heavier than 3-month-old animals, comparing 24-month-old with intermediate ages (6- and 12-month-old mice), we could observe a drop in body weight in both genotypes.

The similar loss of muscle weight in WT and *Ghrl* KO during aging suggested that ghrelin deletion could have no effects on muscle mass. However, a more accurate assessment of muscles uncovered relevant differences between WT and *Ghrl* KO mice. The lack of ghrelin affected muscle cross-sectional areas, with old *Ghrl* KO muscles displaying a reduced number of the smallest myofibers in CSA distribution (Figure 4E) and generally a reduced induction of markers of protein catabolism compared to WT animals (Figure 4A–4C). Of note, the expression of Atrogin-1 was lower also in young *Ghrl* KO mice than in WT littermates (Figure 4A). The protection from age-induced muscle atrophy apparently does not depend on the modulation of IGF-1 that in old *Ghrl* KO mice is even lower than in WT (Figure 4D). On the contrary, the preservation of *Ghrl* KO muscles may partially be due to an increased mitochondrial content in *Ghrl* KO, as suggested by the increased levels of TFAM and COX4 (Figure 5B, 5C).

The changes in muscle mass occurring during aging may arise from the age-related dysregulation of the immune system that leads to progressive systemic inflammation, with a general increase in circulating pro-inflammatory cytokines, which can affect muscle homeostasis. Actually, old WT mice exhibited higher spleen dimension, increased levels of TNF-α expression in epidydimal adipose tissue, and a general tendency of augmented plasmatic pro-inflammatory markers compared to young animals, suggesting the existence of low-grade systemic inflammation (Table 1 and Figure 6A, 6B). Based on the vast amount of the anti-inflammatory activities of ghrelin peptides [31], a worsening of the inflammatory status in *Ghrl* KO mice was plausible. However, in *Ghrl* KO mice a trend towards reduced inflammation could be observed, both in the adipose tissue and in the circulation (Figure 6A, 6B). Such a phenotype closely resembles that of *Ghsr* KO mice [32], where the ablation of AG receptor promotes an anti-inflammatory (M2) macrophages shift, reduced macrophage infiltration, and decreased pro-inflammatory cytokine expression in both white and brown adipose tissues. Also, the pro-inflammatory effects of Ghsr-1a on macrophages profoundly limits lipid mobilization and energy expenditure in adipose tissue [21], processes that are highly perturbed during aging [33]. In particular, it was proposed that AG could participate in the age-related impairment of thermogenesis –thus contributing to the onset of age-related obesity and insulin resistance– through its receptor Ghsr-1a, whose expression in fat increases with aging [32]. Altogether, these observations strongly suggest that, in *Ghrl* KO mice, one of the major determinant factors of their overall better condition is the lack of AG-mediated activation of Ghsr-1a. Conversely, the lack of UnAG in these mice becomes critical under stressful conditions, such as starvation or acute muscle injury, impacting on the physiological homeostasis [9, 22].

Of note, the feeding behavior of *Ghrl* KO mice was different from what previously reported [18, 34], as we observed higher food consumption in young *Ghrl* KO mice (Table 1).

Besides the effects on skeletal muscle, aging also associates often with a gradual cognitive impairment and depression that deeply worst elder people quality of life [35]. The role of AG in regulating sleep and memory [36] and its anti-depressant activity when administered to stressed mice [37] suggest that the impairment of ghrelin signaling during aging could affect cognitive function as well. In assessing the behavioral contribution to the physical performance tests, we skimmed the surface of this issue and surprisingly found that the lack of ghrelin peptides improved the memory capacity of young mice, although this effect was not maintained during aging (Figure 3C). Conversely, the lack of the ghrelin gene protected against the onset of the aging-dependent depression-like behaviors (Figure 3D). Altogether, the pattern of cognitive function could have partially affected the measure of endurance in the hanging wire test (Figure 3A, 3B), as the cleverness could drive the mice to quickly reach the ends of the wire (Figure 3C), while the anxiety/depression (Figure 3D) may induce the mice to abandon the attempt. Therefore, old WT mice could have performed worse than their young counterpart also because of the establishment of age-induced anxiety/depression. On the contrary, *Ghrl* KO mice could have had a starting advantage due to higher cognitive performance in youth. Although this parameter declined with age, it reached the level of young WT animals, and the lack of anxiety/depression during aging could have resulted in overall better performances both in young and old *Ghrl* KO mice than their WT peers.

Exogenous administration of AG to overcome a presumed peripherical ghrelin resistance has been explored in several contexts characterized by loss of skeletal muscle mass, including cancer cachexia [38, 39] and aging [18]. However, while in cancer cachexia the administration of AG has a beneficial activity on skeletal muscle also by contrasting anorexia and the rapid loss of adipose tissue, in sarcopenia the effects on skeletal muscle are limited and accompanied by an increase in adiposity that might worsen age-related insulin resistance and metabolic dysfunction. The pro-adipogenic feature of AG relies on its acylation and resulting binding to Ghsr-1a [6, 40]. Since UnAG does not activate this receptor and does not increase fat mass, while protecting the skeletal muscle from several atrophic stimuli [41], we investigated the potentiality of this hormone in preventing sarcopenia by monitoring the aging of *Myh6/Ghrl* Tg mice characterized by normal levels of AG but increased levels of UnAG [7, 13]. Despite both Tg and WT animals display similar tendencies in how total body weight, epididymal fat, and skeletal muscle mass change with aging (Figure 2 and Table 1), higher circulating levels of UnAG seem to globally maintain in the long run the structure, performance, and metabolism typical of young muscles (Figure 3A, 3B, Figures 4, 5), despite a reduction with aging of *Igf1* expression in muscles (Figure 4D). In particular, old Tg muscles display higher levels of mitochondrial proteins than WT littermates (Figure 5B) that likely accounts for the maintenance of the oxidative metabolism (Figure 5A) and the overall protection from sarcopenia. This is consistent with the ability of UnAG to impair muscle catabolism by mitigating mitochondrial damage [14]. The preserved muscle mass and functionality may be the result of the direct anti-atrophic activity of UnAG [7, 8], as well as of the seeming reduced age-associated chronic inflammation (Figure 6B). Although old Tg spleen weight did not differ from that of WT (Table 1), old Tg animals showed a tendency towards lower levels of inflammatory markers (Figure 6B), such as SDF-1α, I-309, leptin, M-CSF, IL-12p70, and higher levels of soluble TNF-R1 that can act as scavenger for the circulating inflammatory TNF-α [42]. On the other hand, age-associated IL-12 and IL-13 levels were quite similar to those observed in WT animals, possibly reflecting the presence of AG in Tg mice.

To sum up, we showed that both high circulating levels of UnAG and the lack of ghrelin gene prevent the decline of muscle function associated with aging, although with different dynamics. Indeed, old Tg mice seem to preserve the characteristics of young animals, while *Ghrl* KO mice features deteriorate with aging. However, young *Ghrl* KO mice in general show more favorable traits compared to WT animals that result, on the whole, in better performances in aged *Ghrl* KO animals. The only exception is the SDH activity (Figure 5A) that declines with age in both WT and *Ghrl* KO mice, but, differently from the other parameters assessed in this work, young *Ghrl* KO mice do not have the initial advantage of higher SDH activity in youth. On the contrary, similarly to other parameters, Tg mice maintain a constant SDH activity, putatively due to higher levels of mitochondrial proteins (Figure 5B, 5C). Therefore, we can hypothesize that the maintenance of the oxidative metabolism could, at least in part, explain the protection from sarcopenia in Tg mice, while, in *Ghrl* KO mice, the lack of Ghsr-1a-mediated inflammation could play a major role. The fact that *Ghrl* KO mice are protected from sarcopenia might still appear in contradiction with our previous work demonstrating that both AG and UnAG have anti-atrophic activities [7]. However, it should be noted that the anti-atrophic effects of AG and UnAG were assessed in *Ghsr* KO mice, *i.e.*, in circumstances in which AG was not signaling via Ghsr-1a, but through a yet unidentified receptor.

The astonishingly similarity between old *Ghrl* KO mice and old *Ghsr* KO mice indeed strongly suggests a detrimental role for GHSR-1a in the onset of sarcopenia. We reckon that, in mice, the physiological increase of UnAG with aging is not enough to counteract the adverse effects of the increased AG levels. To test this hypothesis, we administered pharmacological doses of UnAG to aging (18-month-old) WT mice for 4 weeks and assessed the effects on muscle mass and performance. As expected, UnAG, which does not signal through Ghsr-1a, did not induce changes in body weight, food intake, and adiposity (Figure 7A–7C). UnAG-treated mice showed higher gastrocnemii and quadriceps muscle weights (Figure 7D) that likely reflect protection from the sharp reduction in muscle mass observed between middle-aged and old mice (Figure 2C and Table 1), rather than induction of hypertrophy. Accordingly, UnAG treatment did not induce changes in *Igf1* expression (Figure 7H). UnAG was able to gradually improve the physical performances of treated mice (Figure 7E, 7F). Remarkably, the higher muscle mass and the better physical performances were not accompanied by a reduction in the expression of genes associated with protein catabolism (Figure 7I, 7J), nor by a shift towards larger fiber areas in muscle sections (Figure 7G). Paradoxically, the CSA distribution showed even a reduction in the frequency of larger fibers in UnAG-treated mice. However, this did not reflect an atrophic status, since there was not an overall shift towards smaller CSA but a rise in the portion of middle-sized fibers. We postulate that this increase in middle-sized fibers could reflect the UnAG-induced shift towards oxidative metabolism (Figure 7K) that is usually characterized by fibers with reduced areas compared to the glycolytic ones.

Altogether, these results suggest that, in humans, the adverse effects of impaired ghrelin signaling in aging could be mainly due to the decline in UnAG rather than AG and should encourage the design of analogs to UnAG rather than AG to therapeutically treat sarcopenia.

## MATERIALS AND METHODS

### Animals and treatments

Animal experiments were performed according to procedures approved by the Institutional Animal Care and Use Committee at the University of Piemonte Orientale. C57BL/6 male mice, matched for age and weight, were used for all experiments. C57BL/6 *Ghrl^-/-^* (*Ghrl* KO) and C57BL/6-*Myh6/Ghrl* transgenic (Tg) mice were generated as previously described [7, 22, 43]. WT mice were littermates of Tg animals. *Ghrl* KO strain was kept in homozygosity. Animals were housed in groups of 4/6 animals and fed *ad libitum* with unrestricted access to drinking water. For food intake analysis and behavioral tests, mice were single-caged for the acclimation plus experimental periods. The light/dark cycle in the room consisted of 12/12 h with artificial light. The numbers of mice ranged from 4 to 27 per experiment. BMI was calculated as animal weight/(nose-to-anus length)^2^. AG and UnAG plasmatic levels were measured by EIA kits (SPIbio Bertin Pharma). In the experiments with i.p. injections of 100 μg/Kg UnAG (PolyPeptide Laboratories; Strasbourg, France), controls were age- and weight-matched saline-injected animals. Mice were killed through cervical dislocation, and tissues used for the study immediately excised, weighted, and processed or stored for further analysis.

### Hanging wire test

A wire-hanging test was employed to assess whole-body muscle strength and endurance. The test was performed as previously described [13]. Briefly, mice were subjected to 180 sec hanging test, during which “falling” and “reaching” scores were recorded. When a mouse fell or reached one of the sides of the wire, the “falling” score or “reaching” score was diminished or increased by 1, respectively. A Kaplan-Meier-like curve was created afterward. Moreover, the longest time between two falls was taken as the latency-to-fall value and used to calculate the holding Impulse (i.e., the hanging time x body mass).

### Object recognition test

Recognition memory was measured using various objects of different sizes and materials, as previously described [44]. Briefly, mice were separated in single cages 10 days before the test. The task started with a familiarization trial (day 1) in which two identical objects were presented to the animals, followed by the test trial (day 2), where one familiar object was substituted with a novel one. In detail, in day 1, mice were left with two identical objects (familiarization phase). Exploration was recorded for 10 min by an investigator blinded to the strain assessed. Sniffing, touching, and stretching the head toward the object at a distance ≤ 2 cm were scored as object investigation. In day 2 mice were again left in the same cage containing two objects: one identical to one of the objects presented during the familiarization phase (familiar object), and a new, different one (novel object), and the time spent exploring the two objects was recorded for 10 min. Memory was expressed as a discrimination index calculated as follows: (seconds on novel–seconds on familiar)/(seconds on novel + seconds on familiar). Animals with memory impairment spend lesser investigating time with the novel object, thus giving a lower discrimination index.

### Tail suspension test

Mice anxiety/depression was measured as previously described [45]. Briefly, mice were individually suspended in the air by tape attached to a shelf (50 cm height) for 6 min. Trials were video-recorded, and a blinded experimenter scored the time during which mice remained immobile as a measure of depressive-like behavior. A small cylindrical tube was slipped over the mouse tail to prevent climbing motion and to escape.

### Histological analysis

Muscles were excised, weighted, mounted in Killik embedding medium (Bio-optica), frozen in liquid-nitrogen-cooled isopentane, and stored at -80 °C. Transverse muscle sections (7 μm) were cryosectioned from the middle part of each muscle. Myofiber areas were determined by immunofluorescence with anti-laminin (1:200; Dako). Slices were fixed in 4% PFA for 20 min, washed, permeabilized with 0.2% Triton X-100 in 1% BSA for 15 min, and blocked with 4% BSA for 30 min. One hour of incubation with primary antibodies was followed by 45 min of secondary antibody (Alexa Fluor 488-anti-rabbit; Thermo Fisher Scientific) at RT. DAPI was incubated for 5 min at RT to visualize nuclei. SDH staining was performed to reveal the oxidative fibers within a muscle. Frozen transverse TA muscles were incubated in a working solution (SUCCINIC DEHYDROGENASE Stain Lyophilized, Bio-optica) for 45 minutes at 37°C. The sections were then rinsed in distilled water, fixed in 4% PFA for 10 minutes, placed in 15% ethanol for 10 minutes, and, finally, mounted with aqueous mounting medium. Images of whole muscle sections were acquired with the slide scanner Pannoramic Midi 1.14 (3D Histech) and cross-sectional areas (CSA) of fibers or SDH staining quantified with ImageJ software (v1.49o).

### Immunofluorescence

Tissue sections were fixed in 4% PFA for 10 minutes, washed in PBS, permeabilized with 1% BSA 0,2% triton x-100 for 20 minutes. For blocking the unspecific binding sites, slices were incubated in 4% bovine serum albumin (BSA) for 2 hours at RT and then sections were stained with an anti-COX4 antibody (1:250; Cell Signaling technology, Danvers, MA, USA) or with an anti-laminin antibody (1:200; Dako, Agilent Technologies, Santa Clara, CA). After washing, sections were incubated with the appropriate Alexa Fluor Dyes-conjugated secondary antibody (488-anti-rabbit; Thermo Fisher Scientific) for 1 hour at RT. 40,6-diamidino-2-phenylindole (DAPI) was incubated for 5 minutes to visualize nuclei.

Images were acquired using the slide scanner Pannoramic Midi Scanner 1.14 (3D Histech) and quantified with ImageJ v1.49o software.

### Gene expression analysis

Total RNA from muscles was extracted by RNAzol (Sigma-Aldrich). RNA was retro-transcribed with High-Capacity cDNA Reverse Transcription Kit (Thermo Fisher Scientific), and real-time PCR was performed with the StepOnePlus Real-Time PCR System (Thermo Fisher Scientific) using the following TaqMan assays: Mm00499518_m1 (*Fbxo32*, Atrogin-1/MAFbx), Mm00439560_m1 (*Igf1*), (Mm00515597_m1 (*Ctsl*), Mm01208835_m1 (*Ppargc1a*), Mm00616415_m1 (*Ghsr*), Mm01225600-g1 (*Bnip3*), Mm00443258_m1 (*Tnf*), and Mm00506384_m1 (*Ppif*).

### Western blotting

Tissue samples from gastrocnemii were homogenized by using GentleMACS Dissociator (Miltenyi Biotec) and lysed in ice-cold RIPA Buffer (50 mM Tris/HCL, pH= 8.0; 1% Triton X-100; 150mM NaCl; 0.5% sodium deoxycholate; 0.1% SDS) supplemented with a protease inhibitor cocktail (Roche) and a phosphatase inhibitor cocktail (Sigma-Aldrich). A clear supernatant was obtained by centrifugation of lysates at 13,000 g for 20 min at 4°C. Protein concentration in the supernatant was determined by Bradford protein assay (Bio-Rad). Aliquots of total cell lysates (20 μg) were then separated by SDS-PAGE by using Miniprotean precast gels (BioRad), and proteins were transferred to nitrocellulose membranes (BioRad).

Membranes were blocked 1h at RT with 5% non-fat milk in Tris-buffered saline with 0.05% Tween 20 and then probed by using the following antibodies directed against PGC1α (AB3242; Millipore), β chain-ATP Synthase (MAB3494; Millipore), PPARγ (sc-7273; Santa Cruz Biotechnology), SDH (sc-377302; Santa Cruz Biotechnology), TFAM (sc-23588; Santa Cruz Biotechnology), GAPDH (G8795; Sigma-Aldrich).

The appropriate secondary horseradish peroxidase-conjugated antibodies from Jackson Immunoresearch were used in 5% not-fat- milk (blocking solution) for 1 h at room temperature. Immunoreactive bands were visualized by Clarity Western ECL Substrate (Biorad). Equal loading of samples was confirmed by GAPDH (G8795; Sigma-Aldrich) normalized and quantified by densitometry by ImageJ Software.

### Mouse inflammation antibody array

Serum cytokines and biomarkers of systemic inflammation were detected using the Mouse Inflammation Antibody Array C1 (RayBiotech, Norcross, Georgia, USA) according to manufacturer instructions.

### Statistical analysis

All data were expressed as mean ± SEM or mean ± SD, when appropriate, absolute values, or percentages. Outliers in the measurements were identified by mean of the interquartile range (IQR), as either below Q1 – 1.5 IQR or above Q3 + 1.5 IQR, and excluded from the analysis. The variation between groups was compared by using nonparametric Wilcoxon, Mann-Whitney U, Friedman, or χ2 tests, as appropriate. Statistical significance was assumed for p<0.05. The statistical analysis was performed with IBM SPSS Statistics for Windows version 25.0. The * symbol indicates a statistically significant difference between old and young animals, for any genotype group; the § symbol identifies a statistically significant difference between WT animals and either Tg or *Ghrl* KO within the same age group.

## Supplementary Material

Supplementary Figures
