# Supplementary material for: Both ghrelin deletion and unacylated ghrelin overexpression preserve muscles in aging mice
Source: Aging (Albany NY). 2020 Jul 26;12(14):13939–57. doi: 10.18632/aging.103802 (PMC7425472; doi:10.18632/aging.103802)
Supplement: Supplementary Figures [file aging-12-103802-s001..pdf]

SUPPLEMENTARY FIGURES

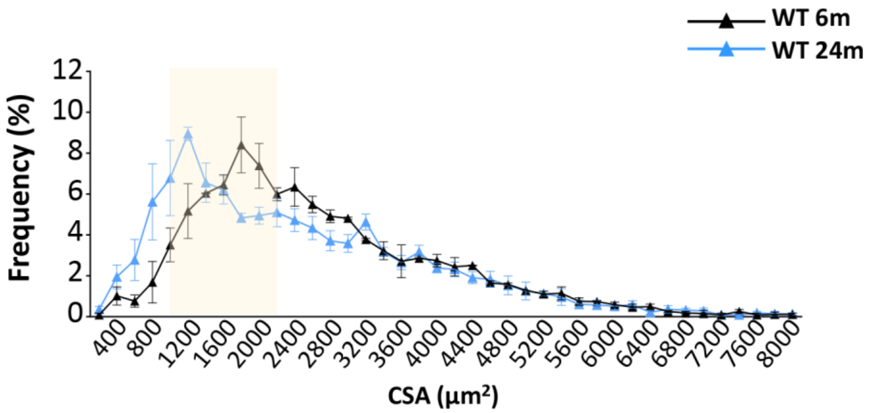

Supplementary Figure 1. Aging-induced atrophy in WT muscles. Cross-sectional area (CSA) frequency distribution of myofibers in GAS of young (N=2) and old (N=4) WT mice. The shadowed area of the graph represents the section of statistically significant differences among curves. Data are presented as mean ± SEM.

	A	B	C	D	E	F	G	H	I	J	K	L
1<br>2	POS	POS	NEG	NEG	BLANK	BLC (CXCL13)	CD30 Ligand (TNFSF8)	Eotaxin-1 (CCL11)	Eotaxin-2 (CCL24)	Fas Ligand (TNFS56)	Fractalkine (TNFSF8)	GCSF (CCL11)
3<br>4	GM-CSF	IFN-gamma	IL-1 alpha (IL-1 F1)	IL-1 beta (IL-1 F2)	IL-2	IL-3	IL-4	IL-6	IL-9	IL-10	IL-12 p70	IL-12 p40/p71
5<br>6	IL-13	IL-17A	I-TAC (CXCL1)	KC (CXCL1)	Leptin	LIX	XCL1	MCP-1 (CCL2)	M-CSF	MIG (CXCL9)	MIP-1 alpha (CCL3)	MIP-1 gamma
7<br>8	RANTES (CCL5)	SDF-1 alpha (CXCL12 a)	I-309 (TCA-3/CCL1)	TECK (CCL25)	TIMP-1	TIMP-2	TNF alpha	TNF RI (TNFRS1A)	TNF RII (TNFRS1A)	BLANK	BLANK	POS

Supplementary Figure 2. Array map of the Mouse Inflammation Antibody Array. Each antibody is spotted in duplicate vertically.
